# Prosocial behavior and youth mental health outcomes: A scoping review protocol

**DOI:** 10.1371/journal.pone.0270089

**Published:** 2022-06-24

**Authors:** Saima Hirani, Emmanuela Ojukwu, Nilanga Aki Bandara

**Affiliations:** 1 School of Nursing, Faculty of Applied Science, The University of British Columbia, Vancouver, Canada; 2 The School of Kinesiology, Faculty of Education, The University of British Columbia, Vancouver, Canada; The Technical University of Kenya, KENYA

## Abstract

**Introduction:**

This review aims to explore the existing literature about the virtue of helping others and its association with youth mental health. Mental health of youth is rooted in their social environment. Helping others or engaging in prosocial behavior are activities that youth may participate in. The notion of helping others and its association with individual mental well-being have been well-studied for adults and older adults and to some extent in youth, however, no review has been conducted to understand the intersection of helping others and mental health in the youth population.

**Methods:**

This review will consider all study designs that examine helping others and mental health of youth. The inclusion criteria for the review will include young individuals aged 10-24-year-old, living in any geographic location, of all gender identities, and with or without mental health issues. Grey literature and studies that only speak to outcomes related to physical well-being will be excluded. A search will be conducted in CINAHL, MEDLINE and PsycINFO. Studies published in the English language will be included with no restriction on publication time period. Articles will be screened against the inclusion criteria onto a single software by two independent reviewers. In the case of any disagreement, a third independent reviewer would resolve the conflict.

**Findings:**

Data will be extracted and presented in a tabular or diagrammatic form supported by a summary. We will report our findings in accordance with the Preferred Reporting Items for Systematic Reviews and Meta-analyses extension for Scoping Reviews (PRISMA-ScR). The findings of this review will provide evidence-based recommendations for promoting youth mental health and a basis for future research.

## Introduction

Young age is a critical stage for mental health over the life course. Over the last several years, youth mental health issues have become a major public health concern and are adding to the burden of youth morbidity and mortality. Worldwide, 10–20% of children and adolescents are affected by mental health disorders with depression and suicide as one of the leading causes of disability and death, respectively [1]. Statistics also indicate that 50% of all psychological conditions start by 14 years of age [1]. The situation is further exacerbated by the fact that many of these adolescents are not receiving the specialized mental health care they require [2]. Evidence suggests that youth who do not receive adequate and timely support may experience poor health consequences in adulthood and limited abilities to lead productive lives [3].

Mental health is deeply rooted in social contexts and belonging [4]. Holistically, mental health and well-being includes the exhibition of positive traits such as positive outlook, strong self-esteem, confidence in one’s ability to cope with adversity, and strong social cohesion [5]. It is well documented in the literature that the social determinants of health pose a notable influence on the psychosocial development of young people [6]. Positive social interactions and approaches offer protective factors for youth mental well-being and reduce their risk for mental health disorders [7]. Evidence also proposes that positive emotional and social connections with others in early human life play a vital role in attaining a better quality of life throughout the life span [8]. Therefore, fostering the positive social development of youth is essential for their overall well-being.

The phenomenon of helping others in formal or informal ways have exhibited positive influences on individuals’ mental health; this virtue has been defined as a voluntary act that aims to benefit and support others [9, 10]. The terms helping others, prosocial behavior, altruism, and volunteerism are used interchangeably in literature [11]. There is a growing focus on exploring this phenomenon among youth. A number of social psychology and developmental theories explain helping others and prosocial attributes as one of the key constructs for positive youth development [11, 12]. There is a consensus that encourages promoting this virtue in young age in order to attain positive psychosocial outcomes in later life [11, 13]. Several studies have reported the positive impact of supporting others on supporters such as reduced depression and stress, improved coping [14], increased happiness [15, 16], higher levels of self-esteem, better academic performance, and stronger social connections [17, 18]. Despite the volume of independent studies in this area, there is limited evidence that synthesizes the understanding of helping others and mental health among youth.

### Aims of the scoping review

A preliminary search of PROSPERO, CINAHL, and the Cochrane Database of Systematic Reviews was conducted and no current or underway scoping reviews on the topic were identified. We identified a systematic review that reported factors contributing to the development of empathy and prosocial behavior in adolescents [19]. However, there is no review that summarizes the scope of knowledge explaining the notion of youth engagement in helping others and its association with their mental health. Therefore, the objective of the proposed review is to present an overview of the extent of evidence related to the virtue of helping others and youth mental health. We aim to identify the existing knowledge and research gap on this topic and recommend directions for future research.

## Methods

Our review will be conducted in accordance with the Arksey & O’Malley’s five stage scoping review framework: (i) identifying a research question, (ii) locate appropriate articles, (iii) study inclusion, (iv) data organization, (v) and result organization and dissemination [20]. The development of this protocol follows the guidelines provided in the Preferred Reporting Items for Systematic Reviews and Meta-Analyses Protocols (PRISMA-P) checklist [21] (S1 Checklist).

### i. Identifying a research question

The proposed scoping review will aim to answer the following question:

What is known from existing literature about the virtue of helping others and its relevance to youth mental health?

### ii. Locate appropriate articles

#### Search strategies for the identification of studies

Our search strategy was developed with the aim of locating published peer-reviewed studies in consultation with a subject expert university librarian. An initial limited search on the topic was undertaken in CINAHL(EBSCOhost). This step helped us to better identify and expand our search terms according to titles, abstracts, and keywords in relevant studies retrieved through this initial search. Subsequently, a full search strategy was developed through multiple refinements of terms for the concept, population, and context in CINAHL(EBSCOhost). Accordingly, this search strategy will be modified and repeated in MEDLINE(Ovid) and PsycINFO databases. The search will be repeated to identify any new citations made available between the initial search date and final manuscript submission. We will contact study authors to request any missing or additional information for clarification. The full initial search strategy is provided in S1 File.

### The search will, in all the databases, consist of the following search terms

1. (MH "Volunteer Experiences") OR (MH "Volunteer Workers")
2. volunteering or volunteerism
3. (MH "Altruism")
4. altruis*
5. "help* others" or prosocial
6. (MH "Service Learning")
7. "service learning"
8. "community service"
9. 1 OR 2 OR 3 OR 4 OR 5 OR 6 OR 7 OR 8
10. (MH "Adolescence+") OR (MH "Young Adult")
11. adolescent* or teen* or youth or "young adult*" or "emerging adult*"
12. 10 OR 11
13. 9 AND 12
14. (MH "Mental Health")
15. (MH "Psychological Well-Being") OR (MH "Wellness")
16. "mental health" or wellness or well-being or resilience or fulfillment or "social anxiety" or contribution
17. (MH "Mental Disorders+")
18. 14 OR 15 OR 16 OR 17
19. 13 AND 18
20. "social participation"
21. 9 OR 20
22. 12 AND 18 AND 21
23. 22 not 19

### iii. Inclusion criteria

#### Types of studies

In this review, studies will not be restricted to a specific study type. We will consider quantitative, qualitative and mixed methods designs for inclusion. Studies published in the English language will be included for this review. No restrictions with regard to publication time will be applied as the review topic is an emerging area of research in youth. Only peer-reviewed material will be included. Grey literature will be excluded. Studies will also be excluded if they report outcomes related to physical well-being only.

#### Focus of studies

This review will include studies exploring the intersection between two broad concepts of helping others and mental health in youth including youth’s experiences, factors related to helping others and their own mental health, impact of helping others interventions on mental health, and relationship between these two concepts. The notion of helping others will be operationalized as any act small or big with the intent to benefit others. The review will also consider studies that have examined a range of other interrelated concepts that express the phenomenon of helping others in relation to youth mental health such as prosocial behaviors, altruism, giving, and volunteerism. The concept of mental health has been defined as a state in which an individual acknowledges their abilities and potentials, effectively handles life stressors, and makes contributions to others [22]. Since mental health is a broad concept and embraces various mental health outcomes and their combination, our review will include all positive mental health variables for e.g. resilience, self-efficacy, stress and coping, self-esteem, social cohesion, quality of life etc. and improvement in mental health issues or negative outcomes such as depression, stress and anxiety etc.

#### Context

This review will include studies that are conducted with youth in any geographical location. Our search will not be limited to any cultural, gender or socioeconomic characteristics. We aim to include all settings in the review including schools, post-secondary institutions, community, hospitals and any voluntary organizations that aim to examine helping others and youth mental health.

#### Participants

This review will consider studies that include young people aged between 10 and 24 years as defined by the World Health Organization (WHO) [23]. This age group also includes adolescents (10–19 years) and youth (15–24 years) categories as classified by the WHO [23]. The population for this review will include young individuals with all gender identities and with or without mental health issues.

#### Study selection

**Fig 1:**
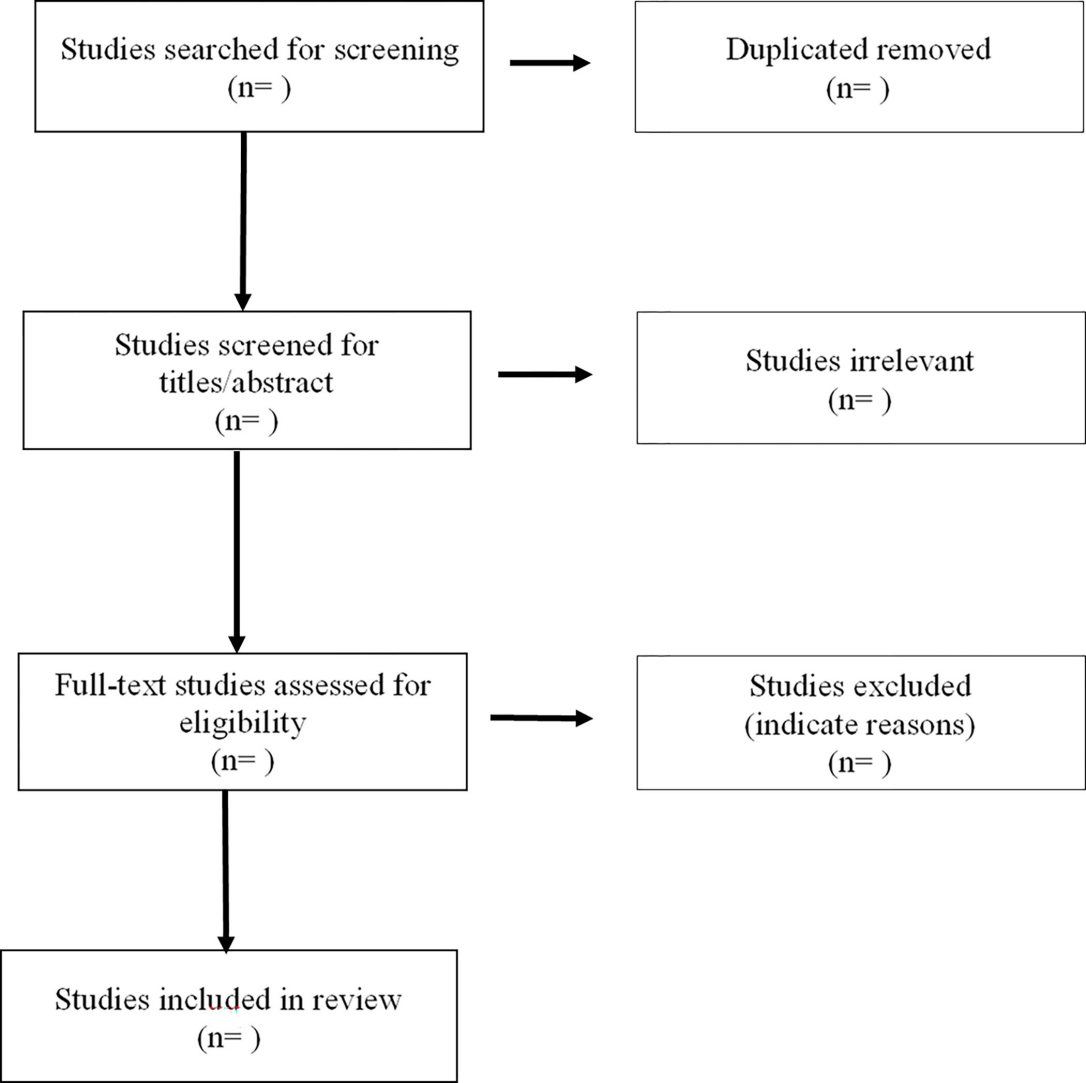
PRISMA flow diagram.

All citations obtained through the three online databases mentioned above will be uploaded into Covidence and duplicates will be automatically removed by the software. Two reviewers will independently review the titles and abstracts and those abstracts that do not match the inclusion criteria will be removed. The full text of the selected citations will be reviewed against the inclusion criteria by two independent reviewers. Exclusion reasons will be recorded for each citation in Covidence in this step. Discrepancies between the reviewers will be resolved by the third independent reviewer and discussion if needed. A flow chart of the screening process will be documented using a PRISMA diagram (Fig 1). The review findings will be reported in accordance with the Preferred Reporting Items for Systematic Reviews and Meta-analyses extension for Scoping Reviews (PRISMA-ScR) guidelines to ensure transparency and reproducibility of the study [24].

### iv. Data organization

#### Data extraction

Data will be extracted from the full papers by two independent reviewers using an extraction tool modified by the reviewers. The data extracted will include specific details about youth (aged 10–24 years), helping others and its association and relevance with mental health outcomes. The draft data extraction tool will be modified and revised as necessary during the process of extracting data from each included paper. Modifications will be detailed in the full scoping review. Any disagreements that arise between the reviewers will be resolved with a third reviewer. Authors of papers will be contacted to request missing or additional data, where required.

### Following categories will be sought in the studies:

1. Authors
2. Title
3. Journal
4. Publication year
5. Country
6. Study objective
7. Study design
8. Context
9. Participant characteristics (Age, gender, sample size)
10. Details/Results extracted from source of evidence (in relation to the concepts of the scoping review)
  - Relationship of helping others and mental health outcomes among youth
  - Youth experiences related to helping others (related concepts) and their mental health outcomes
  - Factors (facilitators and barriers) for youth to engage in helping others for their own mental health
  - Impact of helping others or relevant interventions on youth mental health outcomes/mental well-being

### v. Data synthesis and presentation

Following the PRISMA-ScR guidelines, the number of studies at each stage of the review will be recorded. The studies that are included will undergo thorough data analysis and tabulation.

The extracted data will be presented in a tabular or diagrammatic form supporting the review’s purpose. The extracted data will include the study purpose, type of study (qualitative, quantitative and mixed methods), study population (youth: age and gender), the context helping others was embedded in and its impact on youth mental health. Our results will also include a narrative summary that will accompany the tabulated/diagrammatic findings and explicitly make the connection between our review question and objective.

## Discussion

Promoting youth mental health is one of the most critical priorities for societies. In this scoping review protocol, we discuss the significance of mental health during young age and its impact throughout the life course. The benefits of helping others, prosocial behavior, altruism, and volunteerism are well-documented in the literature for many different age groups, however, a synthesized knowledge on the psychological benefits of these practices for youth is limited. Our review aims to explore the evidence on the concept of helping others and its association with youth mental health outcomes. Findings from this review may identify key research gaps and point us towards novel approaches of supporting youth mental health, this would have broad implications in the social and health care support youth may receive moving forward.

We believe the proposed scoping review will allow us to carry out a scientific inquiry in this important area and provide an overview of the evidence pertinent to helping others and youth mental health. Our review will exclude studies that solely looked at the impacts of helping others on physical health, as our focus is on mental health and mental well-being. Additionally, our review excludes grey literature, we believe this is necessary in order to ensure that the included studies are evidence based and published in peer reviewed journals. We plan to disseminate our findings through a peer- reviewed scoping review paper that highlights the main findings in the literature.

## Supporting information

S1 Checklist PRISMA-P (Preferred Reporting Items for Systematic review and Meta-Analysis Protocols) checklist: Recommended. (DOC)

S1 File Search strategy. (DOCX)
