# Supplementary material for: Prosocial behavior and youth mental health outcomes: A scoping review protocol
Source: PLoS One. 2022 Jun 24;17(6):e0270089. doi: 10.1371/journal.pone.0270089 (PMC9231775; doi:10.1371/journal.pone.0270089)
Supplement: S1 File — (DOCX) [file pone.0270089.s002.docx]

# S2 File. Search strategy

CINAHL (EBSCOhost) Search

| **Search** | **Query** | **Records retrieved** |
| --- | --- | --- |
| S1 | (MH "Volunteer Experiences") OR (MH "Volunteer Workers") | 16,100 |
| S2 | volunteering or volunteerism | 2,506 |
| S3 | (MH "Altruism") | 2,460 |
| S4 | altruis* | 3,734 |
| S5 | "help* others" or prosocial | 3,317 |
| S6 | (MH "Service Learning") | 1,088 |
| S7 | "service learning" | 1,694 |
| S8 | "community service" | 3,104 |
| S9 | S1 OR S2 OR S3 OR S4 OR S5 OR S6 OR S7 OR S8 | 27,680 |
| S10 | (MH "Adolescence+") OR (MH "Young Adult") | 662,454 |
| S11 | adolescent* or teen* or youth or "young adult*" or "emerging adult*" | 722,589 |
| S12 | S10 OR S11 | 722,589 |
| S13 | S9 AND S12 | 3,015 |
| S14 | (MH "Mental Health") | 41,430 |
| S15 | (MH "Psychological Well-Being") OR (MH "Wellness") | 32,894 |
| S16 | "mental health" or wellness or well-being or resilience or fulfillment or "social anxiety" or contribution | 293,764 |
| S17 | (MH "Mental Disorders+") | 589,836 |
| S18 | S14 OR S15 OR S16 OR S17 | 799,153 |
| S19 | S13 AND S18 | 901 |
| S20 | "social participation" | 6,417 |
| S21 | S9 OR S20 | 33,869 |
| S22 | S12 AND S18 AND S21 | 1,426 |
| S23 | s22 not s19 | 525 |
